# Supplementary material for: Immunofluorescence Analysis as a Diagnostic Tool in a Spanish Cohort of Patients with Suspected Primary Ciliary Dyskinesia
Source: J Clin Med. 2020 Nov 9;9(11):3603. doi: 10.3390/jcm9113603 (PMC7695268; doi:10.3390/jcm9113603)
Supplement: Supplementary file 1 [file jcm-09-03603-s001.pdf]

Immunofluorescence analysis as a diagnostic tool in a Spanish cohort of patients with primary ciliary dyskinesia

Supplementary Materials

Table S1: Patients with absence or aberrant distribution of target proteins by immunofluorescence and correlation with other PCD-analysis techniques.

Patient#	YOB/Gender/Origin/ Consanguinity/Family	PICADAR score [1]/PCD symptoms	nNO (nl/min)	Affected IF markers	HSVM	TEM	Genetics	Diagnosis [2] - Next step	Patient# in Baz-Redón *et al.* 2020 [3]
IF-009	2002/M/Caucasian/N	6/Neonatal distress. Chronic rhinitis and wet cough. Sinusitis. Recurrent otitis and hearing loss. Repeated bronchitis and pneumonia. Bronchiectasis. Persistent atelectasis.	5.1	DNAH5-, DNALI1-	Completely immotile	NA	Negative	Highly likely PCD - WES	53
IF-013	2016/M/Caucasian/N	8/Chronic rhinitis and wet cough. Repeated bronchitis. *Situs inversus.*	NA	DNAH5-	Completely immotile	NA	*DNAI2* (c.184-14G>A het. + c.740G>A/p.Arg247Gln het.)	Confirmed PCD *DNAI2*	31
IF-015	2006/M/Caucasian/N	8/Chronic rhinitis and wet cough. Bronchiectasis. *Situs inversus.*	67.7	DNALI1-, GAS8-	Mainly stiff	NA	*CCDC39* (c.610-2A>G hom.)	Confirmed PCD *CCDC39*	6
IF-021	2007/M/Pakistan/Y/sib. of IF-056, IF-057	8/Chronic rhinitis and wet cough. Recurrent otitis. Hearing loss. Repeated bronchitis and pneumonia. Heterotaxy.	6.4	DNALI1-, GAS8-	Mainly stiff	NA	*CCDC40* (c.1416delG/p.Ile473PhefsTer 2 hom.)	Confirmed PCD *CCDC40*	11
IF-024	1991/M/Caucasian/N	6/Neonatal distress. Chronic rhinitis and wet cough. Recurrent otitis and hearing loss. Repeated bronchitis. Bronchiectasis.	14.6	DNAH5-, DNALI1-	Completely immotile	Loss 30% ODA and 70% IDA	Negative	Highly likely PCD - WES	52

IF-025	1964/M/Caucasian/NA	?/Wet cough. Bronchiectasis. Infertility.	112.8	RSPH9-	Stiff	NA	NA	Highly likely PCD - Genetics	
IF-031	1992/F/Caucasian/N	5/Neonatal distress. Chronic rhinitis and wet cough. Repeated penumonia. Bronchiectasis.	10	RSPH9-	Stiff	Loss 40% ODA and 80% IDA	*RSPH1* (c.85G>T/p.Glu29Ter het. + c.275-2A>C het.)	Confirmed PCD *RSPH1*	36
IF-040	2006/F/Moroccan/N	?/Chronic rhinitis and wet cough. Bronchiectasis. *Situs inversus.*	2.3	DNAH5-	Completely immotile	NA	*TTC25* (c.655_659delCTGAC/p.Leu219CysfsTer62 hom.)	Confirmed PCD *TTC25*	43
IF-041	2015/M/Moroccan/Y	3/Chronic rhinitis and wet cough.	NA	RSPH4A-	Circular	NA	*RSPH4A* (c.1453C>T/p.Arg486STer hom.)	Confirmed PCD *RSPH4A*	38
IF-043	2013/F/Pakistan/Y/sib. of IF-044	4/Chronic rhinitis and wet cough. Repeated pneumonia. Persistent atelectasis.	NA	DNAH5-	Completely immotile	ODA defect	*DNAI2* (c.546C>A/p.Tyr182Ter hom.)	Confirmed PCD *DNAI2*	28
IF-044	2006/M/Pakistan/Y/sib. of IF-043	6/Neonatal distress. Chronic rhinitis and cough. Bronchiectasis.	14.2	DNAH5-	Completely immotile	Partial ODA defect	*DNAI2* (c.546C>A/p.Tyr182Ter hom.)	Confirmed PCD *DNAI2*	29
IF-045	2005/F/Caucasian/N	4/Chronic rhinitis and cough. Bronchiectasis. Recurrent otitis. Hearing loss.	17.2	DNAH5-	Completely immotile	Loss 30% ODA and 70% IDA	*DNAH5* (c.4625_4628delGAGA/p.Arg1542ThrfsTer6 het. + c.12706-2A>T het.)	Confirmed PCD *DNAH5*	14
IF-046	2004/F/Caucasian/N	4/Chronic rhinitis and wet cough.	NA	Proximal DNAH5	Subtle defects (disorganized ciliary beat)	NA	*DNAH9* (c.7822-1G>A het. + c.8992C>T/p.Gln2998Ter het.)	Confirmed PCD *DNAH9*	23

IF-047	1970/M/Caucasian/N	?/Chronic rhinitis and wet cough. Sinusitis. Recurrent otitis and hearing loss. *Situs inversus.*	188.1	Proximal DNAH5	Subtle defects (stiff and disorganized ciliary beat)	NA	Negative	Highly likely PCD - WES	
IF-050	2010/F/Moroccan/Y	4/Chronic rhinitis and cough. Bronchiectasis. Recurrent otitis. Lobectomy.	5.4	RSPH4A+, RSPH9-	Stiff and circular	NA	*RSPH9* (c.293_294delTG/p.Val98Glyfs Ter14 hom.)	Confirmed PCD *RSPH9*	40
IF-053	1984/M/Caucasian/N/father of IF-067	10/Neonatal distress. Chronic rhinitis and wet cough. Sinusitis. Recurrent otitis and hearing loss. Repeated bronchitis and pneumonia. Bronchiectasis. *Situs inversus.*	4.7	DNAH5-	Completely immotile	NA	*DNAH5* (c.2575A>T/p.Lys859Ter het. + c.9730G>T/p.Glu3244Ter het.)	Confirmed PCD *DNAH5*	21
IF-055	2011/F/Caucasian/Y	6/Neonatal distress. Chronic rhinitis and wet cough. Recurrent otitis and hearing loss. Repeated bronchitis and pneumoniae. Bronchiectasis.	NA	DNAH5-	Completely immotile	NA	*DNAH5* (3.3kb inc. ex.29 and ex.30 del het.)	Confirmed PCD *DNAH5*	22
IF-056	2018/F/Pakistan/Y/sib. of IF-021, IF-057	7/Neonatal distress. Chronic rhinitis and cough. Recurrent atelectasis.	NA	DNALI1-, GAS8-	Stiff and immotile	NA	*CCDC40* (c.1416delG/p.Ile473PhefsTer 2 hom.)	Confirmed PCD *CCDC40*	12
IF-060	2003/F/Caucasian/N	8/Chronic rhinitis and wet cough. *Situs inversus.*	44.5	DNALI1-, GAS8-	Mainly stiff	ODA and IDA defects	*CCDC40* (c.2T>G/p.Met1Arg het. + 526pb inc. ex.8 and ex.9 del het.)	Confirmed PCD *CCDC40*	13
IF-061	2011/M/Caucasian/N	8/Neonatal distress. Chronic rhinitis and wet cough. Bronchiectasis. *Situs inversus*.	5.5	DNAH5-	Immotile, residual motility	NA	*DNAH5* (3.2kb inc. ex.2 and ex.3 del het. + c.10813G>A/p.Asp3605Asn het.)	Confirmed PCD *DNAH5*	19

IF-062	2002/M/Caucasian/N	8/Chronic rhinitis and wet cough. Sinusitis. Recurrent otitis and hearing loss. Repeated bronchitis. Bronchiectasis. *Situs inversus.*	NA	DNAH5-	Completely immotile	NA	*DNAI2* (c.346-3T>G hom.)	Confirmed PCD *DNAI2*	30
IF-071	1957/F/Caucasian/N	6/Neonatal distress. Chronic rhinitis and sinusitis. Hearing loss. Bronchiectasis.	5.6	GAS8-	Hyperkinetic stiff cilia	NA	NA	Highly likely PCD	
IF-072	2001/M/Caucasian/N	6/Neonatal distress. Chronic rhinosinusitis and wet cough. Bronchiectasis.	NA	DNALI1-, GAS8-	Immotile	NA	*CCDC39* (c.2250delT/p.Gln751LysfsTer11 hom.)	Confirmed PCD *CCDC39*	5
IF-073	2014/M/Caucasian/N	9/Neonatal distress. Wet cough. Recurrent otitis. *Situs inversus.*	NA	DNAH5-	Immotile	NA	*DNAH5* (c.2283_2284del/p.Arg761SerfsTer10 het. + c.3861T>G/p.Tyr1287Ter het.)	Confirmed PCD *DNAH5*	17
IF-076	2004/M/Caucasian/N	3/Chronic rhinitis. Repeated bronchitis. Bronchiectasis.	193.3	Proximal DNAH5	Subtle defects (stiff and immotile)	NA	Negative	Highly likely PCD - WES	
IF-089	2004/M/Caucasian/N	8/Chronic rhinitis. Recurrent otitis. Repeated bronchitis. Bronchiectasis. *Situs inversus.*	27.4	DNAH5-	Completely immotile	NA	NA	Highly likely PCD	
IF-091	2016/F/NA/NA	?/Neonatal distress. Chronic rhinitis and wet cough. Repeated pneumonia. *Situs inversus.*	NA	DNAH5-	Immotile, residual motility	NA	NA	Highly likely PCD - Genetics	
IF-092	2016/M/Caucasian/N	7/Neonatal distress. Chronic rhinitis and wet cough.	NA	DNALI1-, GAS8-	Immotile	NA	*CCDC39* (c.357+1G>C het. + c.2505_2506delCA/p.His835GlnfsTer4 het.)	Confirmed PCD *CCDC39*	4

IF-093	2002/M/NA/NA	?/Neonatal distress. Chronic rhinitis and wet cough. Rhinosinusitis. Recurrent otitis. Repeated pneumonia. Bronchiectasis.	NA	DNAH5-	Immotile, residual motility	NA	NA	Highly likely PCD - Genetics	
IF-094	2011/F/NA/NA	?/Neonatal distress. Chronic rhinitis and wet cough. Recurrent otitis. Bronchiectasis. Persistent atelectasis. *Situs inversus.*	NA	DNAH5-, DNALI1-	Completely immotile	NA	NA	Highly likely PCD - Genetics	
IF-095	2004/F/Caucasian/NA	9/Neonatal distress. Hearing loss. Repeated pneumonia. *Situs inversus*.	NA	DNAH5-	NA	ODA hypoplasia	*CCDC151* (c.410G>A/p.Trp137Ter hom.)	Confirmed PCD *CCDC151*	3
IF-096	1972/M/NA/NA	?/Neonatal distress. Chronic rhinitis and wet cough. Rhinosinusitis. Recurrent otitis. Repeated pneumonia. Fertility problems.	NA	DNAH5-	Immotile	NA	NA	Highly likely PCD - Genetics	
IF-098	2014/M/NA/NA	?/Neonatal distress. Chronic rhinitis and wet cough. *Situs inversus.*	NA	DNALI1-, GAS8-	Immotile and stiff	NA	NA	Highly likely PCD - Genetics	

Patient#: patient identification number; YOB: year of birth; PCD: primary ciliary dyskinesia; nNO: nasal nitric oxide; IF: immunofluorescence; HSVM: high-speed video-microscopy; TEM: transmission electron microscopy; M: male; F: female; N: no; Y: yes; Sib.: sibling; NA: not available data; ?: PICADAR was not calculated due to missing data; +: present in ciliary axoneme; -: absent in ciliary axoneme; ODA: outer dynein arms; IDA: inner dynein arms; het.: heterozygous variant; hom.: homozygous variant; inc.: including; ex.: exon; WES: whole-exome sequencing

Table S2: Patients with normal localization of target proteins by immunofluorescence and correlation with other PCD-analysis techniques.

Patient#	YOB/Gender/Origin/ Consanguinity/Family	PICADAR score [1]/PCD symptoms	nNO (nl/min)	Affected IF markers	HSVM	TEM	Genetics	Diagnosis [2] - Next step	Patient# in Baz-Redón *et al.* 2020 [3]
IF-002	2016/M/Caucasian/N	11/Neonatal distress. Chronic rhinitis and cough. *Situs inversus totalis.*	15.01	All markers +	Hyperkinetic stiff cilia	NA	*DNAH11* (c.12507+1G>C het. + c.13415_13416insCAAA/p.Thr 4472fs het.)	Confirmed PCD *DNAH11*	24
IF-003	2001/F/Caucasian/N	4/Chronic rhinitis and wet cough. Sinusitis. Recurrent otitis and hearing loss. Repeated pneumonia. Bronchiectasis.	3.5	All markers +	Immotile	NA	Negative	Highly likely PCD - WES	48
IF-005	2004/F/South-American/N	?/Chronic rhinitis and wet cough. Sinusitis. Recurrent otitis and hearing loss. Repeated bronchitis and pneumonia. Bronchiectasis.	50.2	All markers +	Disorganized ciliary beat	NA	Negative	Highly likely PCD - WES	47
IF-010	2002/M/Moroccan/NA	?/Chronic rhinitis and wet cough. Recurrent otitis and hearing loss. Bronchiectasis.	13.8	All markers +	Normal	NA	Negative	Highly unlikely PCD	
IF-011	1999/M/Caucasian/N	?/Chronic rhinitis and wet cough. Recurrent otitis and hearing loss. *Situs inversus.*	14.3	All markers +	Stiff	NA	*SPAG1* (c.583delA/p.Ile195Ter het. + c.1855G>C/p.Asp619His het.)	Highly likely PCD – Re-evaluation of variants and/or IF with new sample.	41
IF-016	2013/M/Caucasian/N	2/Chronic wet cough. Bronchiectasis.	NA	All markers +	Normal	NA	Negative	Highly unlikely PCD	
IF-017	2016/M/Moroccan/N	8/Neonatal distres. *Situs inversus.*	NA	All markers +	Normal	NA	Negative	Highly unlikely PCD	

IF-018	1994/M/Caucasian/N	4/Chronic rhinitis and wet cough. Nasal polyps. Sinusitis. Recurrent otitis and hearing loss. Repeated bronchitis and pneumoniae. Bronchiectasis.	14.8	All markers +	Immotile and disorganized ciliary beat	Loss 10% ODA and 40% IDA	Negative	Highly likely PCD - WES	46
IF-022	2003/F/Caucasian/N	2/Chronic rhinitis. Recurrent otitis.	100	All markers +	Normal	NA	Negative	Highly unlikely PCD	
IF-023	2013/F/Caucasian/N	?/Wet cough	NA	All markers +	Normal	NA	Negative	Highly unlikely PCD	
IF-028	2010/F/Caucasian/N	2/Bronchiectasis	NA	All markers +	Normal	NA	Negative	Highly unlikely PCD	
IF-029	2012/F/Caucasian/N	4/Neonatal distress. Repeated bronchitis and pneumonia.	NA	All markers +	Normal	NA	Negative	Highly unlikely PCD	
IF-030	2006/M/Caucasian/N	?/Chronic rhinitis and wet cough. Bronchiectasis.	196.8	All markers +	Normal	NA	Negative	Highly unlikely PCD	
IF-032	1985/M/Caucasian/NA	?/Wet cough. Repeated pneumonia. Bronchiectasis. Infertility.	103.2	All markers +	Reduced CBF	NA	NA	Highly likely PCD - Genetics	
IF-034	2007/F/Caucasian/N	6/Neonatal distress. Chronic rhinitis and wet cough. Recurrent otitis and hearing loss. Repeated bronchitis. Bronchiectasis.	128.7	All markers +	Completely immotile	NA	Negative	Highly likely PCD - WES	44
IF-035	2007/M/Caucasian/NA	?/Wet cough. Bronchiectasis.	NA	All markers +	Normal	NA	Negative	Highly unlikely PCD	
IF-036	2004/M/Caucasian/N	2/Bronchiectasis. Repeated bronchitis and pneumonia.	NA	All markers +	Normal	NA	Negative	Highly unlikely PCD	
IF-037	1987/M/Caucasian/N	?/Chronic rhinitis and wet cough. Nasal polyps. Sinusitis. Repeated pneumonia. Bronchiectasis. Lobectomy.	24.3	All markers +	Immotile	NA	Negative	Highly likely PCD - WES	50

IF-039	2010/F/Caucasian/N	4/Chronic rhinitis and wet cough. Nasal polyps. Recurrent otitis and hearing loss. Bronchiectasis.	15.8	All markers +	Stiff and disorganized ciliary beat	NA	Negative	Highly likely PCD - WES	45
IF-042	2011/F/Caucasian/NA	3/Chronic rhinitis. Bronchiectasis.	NA	All markers +	Normal	NA	NA	Highly unlikely PCD	
IF-048	2005/F/Caucasian/N	7/Neonatal distress. Chronic rhinitis and cough. Recurrent atelectasis.	44.5	All markers +	Normal	NA	Negative	Highly unlikely PCD	
IF-049	2017/M/Arabian/Y	7/Neonatal distress. Chronic rhinitis and wet cough. Repeated bronchitis. Cardiopathy.	NA	All markers +	Hyperkinetic stiff cilia	NA	*DNAH11* (c.983-1G>T het. + c.3439C>T/p.Gln1147Ter het.)	Confirmed PCD *DNAH11*	26
IF-051	2004/M/Caucasian/N	4/Chronic rhinitis and cough. Bronchiectasis. Recurrent otitis. Hearing loss.	NA	All markers +	Normal	NA	NA	Highly unlikely PCD	
IF-052	1963/M/Caucasian/N	3/Chronic rhinitis and cough. Bronchiectasis. Infertility	12.8	All markers +	Mainly stiff	NA	NA	Highly likely PCD - Genetics	
IF-057	2016/M/Pakistan/Y/sib. of IF-021, IF-056	2/Recurrent bronchitis and pneumonias.	NA	All markers +	Normal	NA	Negative	Highly unlikely PCD	
IF-058	1964/F/Caucasian/N	2/Bronchiectasis. Recurrent pneumonias.	218.7	All markers +	Normal	NA	Negative	Highly unlikely PCD	
IF-059	2002/F/Caucasian/N	2/Recurrent pneumonias	300	All markers +	Normal	NA	Negative	Highly unlikely PCD	
IF-063	1967/F/Caucasian/NA	?/Chronic rhinitis and wet cough. Sinusitis. Recurrent otitis and hearing loss. Repeated bronchitis. Bronchiectasis. *Situs inversus.*	11.4	All markers +	Stiff and immotile	NA	Negative	Highly likely PCD - WES	49

IF-064	2016/F/Caucasian/N	3/Chronic rhinitis. Persistent atelectasis.	NA	All markers +	Normal	NA	NA	Highly unlikely PCD	
IF-066	1983/F/Caucasian/N	8/Chronic rhinitis and cough. Recurrent otitis. Hearing loss. Neonatal distres and intensive care	154.7	All markers +	Normal	NA	NA	Highly unlikely PCD	
IF-067	2018/M/Caucasian/N/son of IF-053	2/Recurrent bronchitis	NA	All markers +	Normal	NA	Negative	Highly unlikely PCD	
IF-068	2000/M/Caucasian/N	4/Chronic rhinitis and wet cough. Sinusitis. Recurrent otitis and hearing loss. Repeated bronchitis. Bronchiectasis.	4.3	All markers +	Hyperkinetic stiff cilia	NA	*DNAH11* (c.3898C>T/p.Gln1300Ter het. + c.6983+1G>A het.)	Confirmed PCD *DNAH11*	27
IF-077	2011/F/Moroccan/NA	2/Bronchiectasis	145	All markers +	Normal	NA	NA	Highly unlikely PCD	
IF-097	1973/F/NA/NA	?/Chronic rhinitis and wet cough. Recurrent otitis and hearing loss. Bronchiectasis. Fertility problems.	NA	All markers +	Immotile and stiff	NA	NA	Highly likely PCD - Genetics	
IF-102	2016/M/Pakistan/Y	2/Recurrent bronchitis	44.2	All markers +	Disorganized ciliary beat and stiff	NA	NA	Highly likely PCD - Genetics	

Patient#: patient identification number; YOB: year of birth; PCD: primary ciliary dyskinesia; nNO: nasal nitric oxide; IF: immunofluorescence; HSVM: high-speed video-microscopy; TEM: transmission electron microscopy; M: male; F: female; N: no; Y: yes; Sib.: sibling; NA: not available data; ?: PICADAR was not calculated due to missing data; +: present in ciliary axoneme; -: absent in ciliary axoneme; CBF: ciliary beat frequency; ODA: outer dynein arms; IDA: inner dynein arms; het.: heterozygous variant; WES: whole-exome sequencing

Table S3: Patients with inconclusive and/or insufficient immunofluorescence results and correlation with other PCD-analysis techniques.

Patient#	YOB/Gender/Origin/ Consanguinity/Family	PICADAR score [1]/PCD symptoms	nNO (nl/min)	Affected IF markers	HSVM	TEM	Genetics	Diagnosis [2] – Next step	Patient# in Baz-Redón *et al.* 2020 [3]
IF-006	2004/M/Caucasian/N	8/Chronic rhinitis and wet cough. Bronchiectasis. *Situs inversus*.	14.6	Insufficient and inconclusive	Stiff and immotile	NA	*CCDC39* (c.216_217delTT/p.Cys73GlnfsTer6 het. + c.357+1G>C het.)	Confirmed PCD *CCDC39*	8
IF-012	1977/F/Caucasian/NA	?/Chronic rhinitis and wet cough. Sinusitis. Recurrent otitis. Bronchiectasis.	155.9	Insufficient	Normal	NA	NA	Highly unlikely PCD	
IF-014	2008/M/Caucasian/N	8/Neonatal distress. Repeated bronchitis. *Situs inversus.*	115.8	Inconclusive	Normal	NA	NA	Highly unlikely PCD	
IF-019	2006/M/Caucasian/NA	2/Chronic wet cough. Nasal polyps.	NA	Insufficient and inconclusive	Normal	NA	NA	Highly unlikely PCD	
IF-074	2019/F/Pakistan/Y	9/Neonatal distress. Chronic rhinitis. *Situs inversus.*	9	Inconclusive	Completely immotile	NA	NA	Highly likely PCD - Genetics	
IF-100	2004/M/Caucasian/N	?/Repeated bronchitis.	NA	Insufficient	Normal	NA	NA	Highly unlikely PCD	

Patient#: patient identification number; YOB: year of birth; PCD: primary ciliary dyskinesia; nNO: nasal nitric oxide; IF: immunofluorescence; HSVM: high-speed video-microscopy; TEM: transmission electron microscopy; M: male; F: female; N: no; Y: yes; Sib.: sibling; NA: not available data; ?: PICADAR was not calculated due to missing data; +: present in ciliary axoneme; -: absent in ciliary axoneme; het.: heterozygous variant

References:

1. Behan, L.; Dimitrov, B.D.; Kuehni, C.E.; Hogg, C.; Carroll, M.; Evans, H.J.; Goutaki, M.; Harris, A.; Packham, S.; Walker, W.T.; et al. PICADAR : a diagnostic predictive tool for primary ciliary dyskinesia. *Eur. Respir. J.* **2016**, *47*, 1103–1112, doi:10.1183/13993003.01551-2015.

2. Lucas, J.S.; Barbato, A.; Collins, S.A.; Goutaki, M.; Behan, L.; Caudri, D.; Dell, S.; Eber, E.; Escudier, E.; Hirst, R.A.; et al. European Respiratory Society guidelines for the diagnosis of primary ciliary dyskinesia. *Eur. Respir. J.* **2017**, *49*, doi:10.1183/13993003.01090-2016.

3. Baz-Redón, N.; Rovira-Amigo, S.; Paramonov, I.; Castillo-Corullón, S.; Cols Roig, M.; Antolín, M.; García Arumí, E.; Torrent-Vernetta, A.; de Mir Messa, I.; Gartner, S.; et al. Implementation of a Gene Panel for Genetic Diagnosis of Primary Ciliary Dyskinesia. *Arch. Bronconeumol.* **2020**, *20*, 30073–9, doi:10.1016/j.arbres.2020.02.010.
